# The Gut-Microglia Connection: Implications for Central Nervous System Diseases

**DOI:** 10.3389/fimmu.2018.02325

**Published:** 2018-10-05

**Authors:** Yiliang Wang, Zhaoyang Wang, Yun Wang, Feng Li, Jiaoyan Jia, Xiaowei Song, Shurong Qin, Rongze Wang, Fujun Jin, Kaio Kitazato, Yifei Wang

**Affiliations:** ^1^Guangzhou Jinan Biomedicine Research and Development Center, Institute of Biomedicine, College of Life Science and Technology, Jinan University, Guangzhou, China; ^2^Key Laboratory of Virology of Guangzhou, Jinan University, Guangzhou, China; ^3^Key Laboratory of Bioengineering Medicine of Guangdong Province, Jinan University, Guangzhou, China; ^4^Key Laboratory for Major Obstetric Diseases of Guangdong Province, Department of Obstetrics and Gynecology, Third Affiliated Hospital of Guangzhou Medical University, Guangzhou, China; ^5^College of Pharmacy, Jinan University, Guangzhou, China; ^6^Integrated Chinese and Western Medicine Postdoctoral Research Station, Jinan University, Guangzhou, China; ^7^Division of Molecular Pharmacology of Infectious Agents, Department of Molecular Microbiology and Immunology, Graduate School of Biomedical Sciences, Nagasaki University, Nagasaki, Japan

**Keywords:** brain, central nervous system diseases, gut microbiome, microglia, gut-microglia connection

## Abstract

The importance of the gut microbiome in central nervous system (CNS) diseases has long been recognized; however, research into this connection is limited, in part, owing to a lack of convincing mechanisms because the brain is a distant target of the gut. Previous studies on the brain revealed that most of the CNS diseases affected by the gut microbiome are closely associated with microglial dysfunction. Microglia, the major CNS-resident macrophages, are crucial for the immune response of the CNS against infection and injury, as well as for brain development and function. However, the current understanding of the mechanisms controlling the maturation and function of microglia is obscure, especially regarding the extrinsic factors affecting microglial function during the developmental process. The gut microflora has been shown to significantly influence microglia from before birth until adulthood, and the metabolites generated by the microbiota regulate the inflammation response mediated by microglia in the CNS; this inspired our hypothesis that microglia act as a critical mediator between the gut microbiome and CNS diseases. Herein, we highlight and discuss current findings that show the influence of host microbiome, as a crucial extrinsic factor, on microglia within the CNS. In addition, we summarize the CNS diseases associated with both the host microbiome and microglia and explore the potential pathways by which the gut bacteria influence the pathogenesis of CNS diseases. Our work is thus a comprehensive theoretical foundation for studies on the gut-microglia connection in the development of CNS diseases; and provides great potential for researchers to target pathways associated with the gut-microglia connection and overcome CNS diseases.

## Background

Microglia—the major brain-resident macrophages—are involved in a myriad of processes as the first line of defense against injury and infections, including brain development, brain function, and immune response, in the central nervous system (CNS) (1). Consistent with these diverse roles, microglial dysfunction has been shown to be involved in the initiation or progression of multiple CNS diseases, including Alzheimer's disease (AD), Parkinson's disease (PD), and even autism spectrum disorder (ASD) and depression (2–7). Despite their critical role, their regulatory dynamics during brain development are poorly understood. Several intrinsic factors, such as the fractalkine receptor CX3C chemokine receptor (CX3CR1), MAF BZIP transcription factor B (MafB), and molecules of the complement system, have been found to control the function of microglia (1, 5, 8, 9). However, our understanding of the mechanisms that regulate the maturation and function of microglia *in vivo* is limited, especially those involving extrinsic signals, such as those from the gut microbiome. Indeed, the mammalian microbiome is a signal that integrates environmental cues.

The gut microbiome has been showed to be a crucial signal for multiple biological processes, especially in maintaining the function of CNS, including brain circuitry, neurophysiology, and behavior (10–12). Indeed, the gut-brain axis is a crucial connection associated with multiple CNS diseases (11, 13, 14). The most comprehensive and typical example of this is the generation of host serotonin. In detail, the gut microbiota has been suggested to regulate the biosynthesis of host serotonin, affecting multiple aspects of host behavior in mice and humans (15–17). Furthermore, the gut microbiota has been shown to regulate the permeability of the blood-brain barrier (BBB) (18, 19). Moreover, a recent study has identified a lifelong close correlation between the brain metabolome and the intestinal microbiome in rats (20). Nevertheless, the detailed mechanisms linking the host microbiome and specific CNS diseases are poorly understood. Gut dysbiosis was identified as a crucial factor in animal models of AD and ASD (11, 13); furthermore, germ-free (GF) mice exhibited an excessive stress response (10, 21). These results suggested that several factors significant for CNS dysfunction may be controlled or generated by the gut microbiome. Indeed, in addition to acting as the regulators of peripheral immune function, studies have revealed that the microbiome controls the maturation and immune response of microglia (1, 12, 22, 23). Given that microglia are crucial for CNS immune response, the prior notion that the brain is “immune-privileged” is losing traction (24).

Notably, most microbiome-associated CNS diseases are closely related to the dysfunction of microglia, including AD, PD, MS, depression, and ASD (11, 23), suggesting that microglia may be a potential mediator that link the host microbiome with CNS diseases; however, confirming this hypothesis requires further studies to be conducted. Depression is a microglia-associated disorder because microglial dysfunction is often observed in the brains of patients suffering from depression (25). In addition, the gut microbiome has been suggested to be associated with depression in rodent models (26–29). Moreover, several specific metabolites, generated by the gut microbiome, control the inflammatory response of astrocytes in the experimental autoimmune encephalomyelitis (EAE) mouse model of multiple sclerosis (MS) by activating microglia (23). Collectively, microglia may be crucial mediators in the interaction between CNS diseases and the gut microbiota.

However, the detailed mechanisms controlling the maturation of microglia *in vitro* and *in vivo* remain uncertain; in particular, the mechanisms through which the microbiome affects microglia are only partially understood. Therefore, a comprehensive investigation of the regulation of signaling between microglia and the microbiome may uncover information with tremendous clinical implications for the treatment of several CNS diseases. In this review, we highlight and discuss the recent findings, showing how the host microbiome acts as a crucial environmental factor to control the function and maturation of microglia from before birth until adulthood. In addition, we describe several CNS diseases associated with both the microbiome and microglia in animal (mainly focused on mice) and humans. Our work, thus, provides a comprehensive theoretical foundation for the role of the gut microbiome in the function of microglia; this will provide a more comprehensive understanding of the role of gut flora, as extrinsic signals, in regulating CNS diseases via modulating the maturation and responses of microglia within the CNS.

## Microglia strongly affect CNS architecture and function

Microglia are major players in maintaining the health of the CNS, controlling embryonic wiring, and regulating cell death and survival (5, 30–33). During prenatal development, microglia are the first glial cells to migrate to the CNS, where they participate in the formation of CNS architecture, including neurogenesis, synapse shaping, and excitotoxicity prevention (4), as summarized in Table 1.

**Table 1:** Major functions of microglia in maintaining health in the CNS.

**Function**	**Microglia related process**	**Mediators**	**References**
Neurogenesis	Guiding neurons and axons during the formation of neural circuits in prenatal development	-	(34)
	Integral components in neurogenic niches	CXCL12; CXCR4; ATP	(35, 36)
	Elimination of apoptotic neural stem cells and excess newborn progenitor cells	TAM; Gas6; Protein S	(37, 38)
	Age-associated reduction in neural stem cell proliferation	Proinflammatory cytokines secreted by microglia	(39)
Shaping Synapses	Synaptic pruning or connectivity	C1q; C3; CR3 (Microglia); CX3CR1 (Microglia); CX3CL1	(40, 41) (42, 43)
	Synaptic plasticity	Proinflammatory cytokines; ROS; NO; Neurotrophic factors; BDNF; IL-1β	(44, 45)
	Synaptic transmission	Glutamate; NADPH oxidase; NADPH receptor; PP2A; AMPA receptor;	(46)
	Synapse activity during neuropathic pain transmission	ATP; BDNF; Trk; KCC2 (Cation-chloride cotransporter KCC2)	(47)
Excitotoxicity prevention	Protection against NMDA-induced toxicity	ATP; P2X7; TNF-α	(48)

*CXCL12, C-X-C motif chemokine 12; CXCR4, C-X-C chemokine receptor type 4; TAM, Tyro3, Axl, and Mertk receptors; Gas6, Growth arrest–specific 6; C1q, Complement component 1q; C3, Complement component 3; CR3, Complement receptor 3; CX3CR1, CX3C chemokine receptor 1; CX3CL1, C-X3-C motif chemokine ligand 1; PP2A, Protein phosphatase 2A; AMPA,α-amino-3-hydroxy-5-methyl-4-isoxazolepropionic acid; BDNF, Brain-derived neurotrophic factor; Trk, Neurotrophic receptor tyrosine kinase; P2X7, P2X purinoceptor 7; TNF-α, Tumor necrosis factor alpha*.

Macrophages reprogram their functions in response to pathogens, tissue damage, and lymphocyte interactions through polarization (49). As the major brain-resident macrophages, microglia also have the capacity to be polarized to M1-like or M2-like monocytes (4). Notably, phagocytic M2 phenotype microglia are sub-classified into M2a, M2b or M2c in the absence of inflammation (50) and induce a Th2-like response, M1 and M2 represent a spectrum of activation patterns and not separate cell subtypes. In general, M1-polarized microglia exert cytotoxic effects on specific cells *in vitro*, such as neurons and oligodendrocytes, whereas M2-polarized cells promote neurite outgrowth, exhibit phagocytic capacity (51–53), and support CNS remyelination by driving oligodendrocyte differentiation (54). However, overlapping phenotypes that co-expressed M1 and M2 markers were identified in most human inflammatory and neurodegenerative diseases; therefore, there is limited evidence on microglia polarization in these diseases (55, 56). Nevertheless, substantial evidence has demonstrated that microglia play multiple roles in homeostatic and pathological conditions of the CNS, similar to other tissue-resident macrophages (4). Besides, microglia are the first line of defense against extrinsic microbial infection and injury in the nervous system (22, 57). To perform this role, microglia continuously probe the peripheral environment using a series of receptors and signaling molecules collectively known as sensome genes, which are primarily expressed by the so-called “resting” microglia with high motility (58, 59). Once injury signals are sensed, resting microglia are activated and quickly move toward the indicated sites (60, 61). The phenotypes of these reactive microglia are different, depending on the specific signal and action of the modulators that activated the microglia (62). For instance, when the CNS was infected with the herpes simplex virus (HSV)-1, microglia were identified as the major source of HSV-1-induced chemokine production, including that of CXC ligand 10 (CXCL10), CC ligand 2 (CCL2), and CXCL9, all of which led to peripheral immune cell infiltration into the brain (63). In contrast, HSV-1 infection in CNS led to prolonged activation of microglial cells and retention of T lymphocyte, suggesting that microglia activation contributes to the neuropathological sequelae observed in herpes encephalitis patients (64). However, this does not mean that microglial activation is always beneficial for maintaining the health of the host CNS (65). Indeed, mounting evidence indicated that microglial overactivation contributes to neuronal damage in neurodegenerative diseases. Particularly, reactive oxygen species (ROS), generated from overactivated microglia in response to certain environmental toxins and endogenous proteins, causes neurotoxicity (65, 66).

Given the dual and critical roles of microglia in the CNS, the regulatory mechanisms of microglia are now a major research focus. Microglia originate from hematopoietic stem cells in the yolk sac, and are the first neurons generated at approximately embryonic day 9.5 (E9.5) in mice (9); they then expand and self-renew during adulthood and differentiate to play multiple crucial functions in the CNS (4, 9, 32, 67). The detailed transcriptional program controlling the differentiation process remains unclear, although several factors showed a potential role in their differentiation. *Spi1* (encoding PU.1), *Csf1r*, and interferon regulatory factor 8 (*IRF8*) are the primary microglial transcription and survival genes, all of which are essential for the development of microglia from erythromyeloid progenitors in the yolk sac in mice (8, 9, 68).

## Gut microbiota affects prenatal and adult microglia

Our current *in vitro* and *in vivo* knowledge of the mechanisms controlling microglia is limited. There is a critical gap in our understanding of the environmental factors that control the maturation and function of microglia *in vivo*. The gut microbiota is most associated with extrinsic signals, such as life style, regional strain pools, and has gained considerable attention from researchers, because the human intestine contains a wide range of microbial cells with major roles in multiple host biological processes, including food digestion and pathogen invasion blocking, to maintain general good health (10–12). Moreover, the gut flora influences multiple peripheral immune cells (69) and modulates hematopoietic stem cells and myeloid precursors in the bone marrow to maintain systemic populations of neutrophils in circulation (70). Bone marrow-derived splenic macrophage and monocyte populations were also found to be reduced in GF mice compared with controls (71). Of note, the microbiome appears to play a role in CNS diseases, along with microglia, as discussed in the following section, suggesting a potential association between microbiota and microglia in the development of CNS diseases.

Indeed, the host gut microbiota has been suggested to control the maturation and function of microglia (1, 12, 22, 72). An experimental demonstration of the association between microglia and the microbiome was first performed by Erny et al. (22), who were inspired by numerous studies on the interactions between the CNS and the gastrointestinal system. In their study, GF mice displayed significant microglial defects, with altered cell proportions and an immature phenotype, including more segments, longer processes, and greater numbers of branching and terminal points. This defective microglia phenotype was also observed in other mice with altered microbiota, including the altered Schaedler flora (ASF) mice, which harbored only three bacterial strains (73), and the acute microbiome-depleted mice, following treatment with short-term broad-spectrum antibiotics. Collectively, microglia in conventionally colonized mice would require continuous input from the gut microbiome; this input appeared to be associated with bacterial complexity. In that study (22), treatment with antibiotics failed to increase the number of microglia, in contrast with the effect observed in adult GF mice. Moreover, the expression of *Ddit4*, a regulator of cell growth, proliferation, and survival, was markedly elevated in the microglia of adult GF mice; in contrast, *Ddit4* expression was unaffected in antibiotic-treated mice (Table 2). Several reasons may contribute to the different effect of altering host microbiota between antibiotics-treated mice and GF mice. Firstly, microglia originate from the yolk sac, and their proliferation in adult mice was observed solely in brain-resident microglia (9). The GF mice lacked gut microbiota since birth; it is possible that the host microbiome influences prenatal microglia formation, thus contributing to the emergence of defective microglia in adult mice, which has already been confirmed in a later study (12). Secondly, the antibiotics utilized in the study (22) may have directly targeted microglia in the brain in a microbiota-independent manner. Indeed, several antibiotics, such as minocycline, have been shown to cross the BBB and regulate the activity of microglia, independent of the abundance of the microbiota (75). Furthermore, topical application of aminoglycoside antibiotics has been shown to enhance the host's innate immune response against viral infections in a microbiota-independent manner (76). Of the antibiotics used in that study (22), only metronidazole was able to cross the BBB (77). Therefore, further research is required to test whether treatment with metronidazole only can affect microglial function independent of the abundance of the gut microbiome. Thirdly, Iba1 was selected as a specific indicator of microglia in their study (22); however, it is not an ideal indicator for microglia because multiple myeloid cells are known to express Iba1, including perivascular cells, choroid plexus macrophages, and meningeal macrophages (78), thus, a more specific marker, such as P2Y12, is required to label microglia to analyze their abundance and morphology (79). Therefore, the observed reduction in Iba-1^+^ parenchymal microglia in the CNS of GF mice, which was not observed in the antibiotic-treated mice, may have been due to the label of Iba1 in other myeloid cells within the brain.

**Table 2:** Dominant microglial factors in GF and ABX-treated mice at 6–10 weeks of age.

**Microglia-associated factors**	**Function of indicated factor (References)**	**GF**	**ABX**
*Spi1* (encodes PU.1)	Central microglial transcription and survival factor (68)	↑	-
*Ddit4*	Activation of cell proliferation (74)	↑	→
*Csf1r*	Central microglial transcription and survival factor (8)	↑	-
CSF1R	Surface factors of microglia downregulated during maturation (22)	↑	-
F4/80	As above (22)	↑	↑
CD31	As above (22)	↑	→

*Ddit4, DNA damage-inducible transcript 4; Spi1, Spleen focus forming virus (SFFV) proviral integration oncogene; Csf1r, Colony stimulating factor 1 receptor; F4/80, adhesion G protein-coupled receptor E1 (Aliases); CD31, Platelet/endothelial cell adhesion molecule 1 (Aliases); GF, germ-free; ABX, antibiotics; ↑ means upregulation, → means unaltered, and – means uncertain*.

Moreover, the consequence of defective microglia on viral genes expression and virus loading in the CNS of GF mice or antibiotic-treated mice was not determined as the researchers only assessed the immune response of microglia under GF conditions by analyzing the levels of several immune response genes after exposing the animals to either bacteria or lymphocytic viruses (22). However, microglia are the first line of defense against extrinsic microbial infection in the CNS; thus, the final functional consequences of microglial malformation and immaturity under GF conditions or after antibiotic treatment require further study.

**Figure 1:**
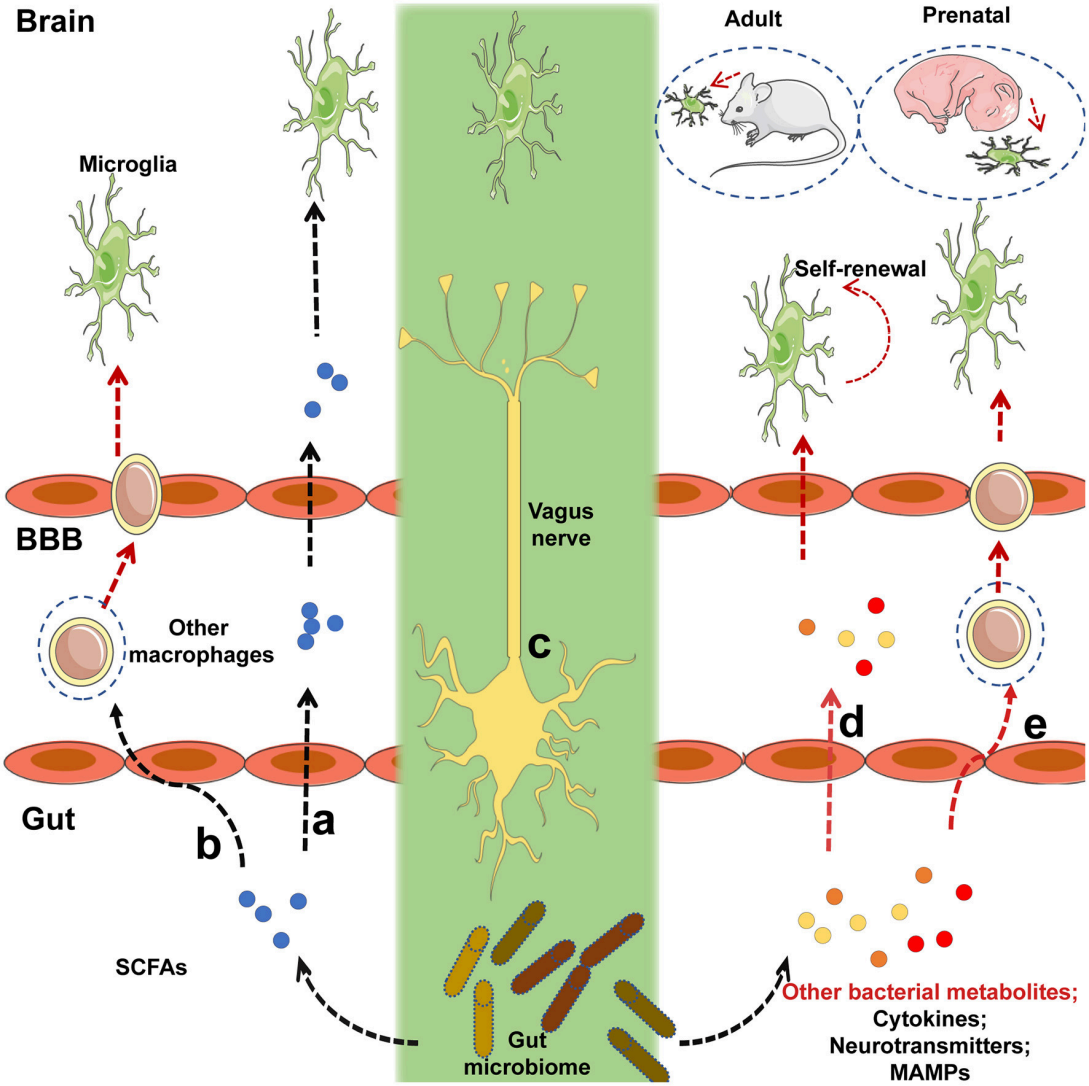
Potential mechanisms by which intestinal microbiota regulate the maturation and function of microglia. **(a)** Short-chain fatty acids (SCFAs) generated by the gut microbiota cross the blood-brain barrier (BBB) via the circulatory system of the host, and target microglia to regulate their function or maturation. **(b)** Immune cells expressing receptors that recognize SCFAs can migrate to the brain via the BBB after signaling by SCFAs that originate from the gut flora. **(c)** The gut microbiota may communicate directly with brain-resident microglia via the vagus nerve. **(d)** Before receptors recognizing SCFAs are expressed, other bacterial metabolites or microbe-associated molecular patterns (MAMPs) generated by the gut microbiota can cross the BBB and target microglia to regulate their function or maturation. **(e)** Peripheral macrophages that can recognize the relevant metabolites or MAMPs can migrate to the brain via the BBB after receiving signals from bacterial metabolites or MAMPs released by the gut flora. Black lines indicated that the corresponding pathways were recognized in a prior study, and red lines represented uncertain pathway. Black lines represent known pathways and red lines indicated uncertain pathways.

Furthermore, the detailed mechanism underlying the regulation of microglia by the gut microbiota requires further research. Although microglia express many pattern-recognition receptors that recognize microbial-associated molecular patterns (MAMPs), no changes in microglia morphology and maturation were found in the absence of several MAMPs, which were recognized by toll-like receptor 3 (TLR3), TLR7, and TLR9 (22), suggesting that commensal microbes may regulate the function of microglia in a MAMP-independent manner. Finally, it has been indicated that short-chain fatty acids (SCFAs) produced by the microbiota through the catabolism of complex carbohydrates (22, 80), are involved in the regulation of microglia maturation (12). Given that GF mice exhibited an increased permeability of the BBB (18) and that SCFAs have been shown to cross the BBB (81, 82), it was not surprising that SCFA administration largely restored microglial activity under GF conditions (Figure 1). Current receptors known to recognize SCFAs, including FFAR2 [also called G protein-coupled receptor (GPR) 43], FFAR3 (also called GPR 41), and GPR109A, are expressed by multiple immune cells and intestinal epithelial cells (80). However, FFAR2 is not expressed in any adult brain cell, although the microglia of mice lacking FFAR2 exhibited defects and the receptors were not essential for SCFA to enter the CNS (83–85). Of note, microglia activity can be modulated by receptors of proinflammatory and anti-inflammatory cytokines in the CNS and in circulation; SCFAs are crucial regulators of nuclear factor-κB (NF-κB) activity and proinflammatory innate immune responses (80, 86). Therefore, in the absence of a receptor expressed on the microglia, that can recognize SCFAs, the possibility that other metabolites from the gut microbiota directly regulate microglia cannot be excluded. SCFAs may control the activity of microglia through other peripheral myeloid cells that express FFAR2, recognize signals from SCFAs, and migrate to the brain or secrete specific factors that can cross the BBB to regulate microglia within the CNS (Figure 1). Propionate, a major SCFA, exhibited protective effects on the BBB by recognizing FFAR3 on the surface of endothelial cells in a recent study (19), indirectly supporting the notion that SCFAs, as gut-derived microbial metabolites, may be crucial mediators in the gut-brain connection. Other bacterial metabolites or MAMPs generated by the gut microbiota may cross the BBB and directly regulate microglial function (Figure 1), especially when the receptors that recognize SCFAs are not yet expressed, given that microbiota may control the microglia before birth (12). Peripheral macrophages that can recognize target metabolites or MAMPs can cross the BBB and migrate to the brain after receiving signals such as bacterial metabolites or MAMPs generated by gut flora (Figure 1). The gut can connect directly to the CNS through the vagus nerve, and the modulation of microglia through external vagus nerve stimulation was found in a murine model of AD (87, 88); the role this link plays in the gut-microglia connection remains uncertain (Figure 1). Further study is warranted to determine the signaling pathways of the gut microbiome that ensure the maturation and function of microglia. Despite these unresolved questions, this study provided evidence that the gut microbiome is closely associated with the maturation of microglia, and that SCFAs are crucial mediators of the association between the gut microbiome and microglia.

In addition to affecting the microglia of adult mice, the maternal microbiome has emerged as a significant regulator of prenatal microglia in a sex-specific manner (12); this is not surprising as the male and female microglia behave differently during the embryonic phase. Under normal conditions, for pain perception, microglia in rodents exhibited sexually dimorphic properties and showed differences in colonization rates in males and females (89–92). Based on data from RNA-sequencing (RNA-seq), the increased expression of genes in microglia from E18.5, and in adult female mice, was associated with the apoptotic process, response to lipopolysaccharides (LPS), and inflammatory response (12, 92). This suggests that microglia were in a more immune-activated state in females; this is in line with a previous study, illustrating that females showed stronger innate and adaptive immune responses (93). In that study (12), the microglia from GF mice exhibited increased ramification and production during the embryonic stages, which is based on the fact that no other differences were observed in the development of embryos from GF mice and specific-pathogen-free (SPF) controls. Under GF conditions, the microglia of male mice were affected more strongly *in utero*; in the adult phase, the host microbiota exhibited a more profound effect on the microglia of female mice. Transcriptome data indicated that 1,216 differentially expressed genes (DEGs) were observed in E18.5 male microglia between control and GF embryos, while only 20 DEGs were found in female microglia at the same phase. These DEGs included immune response-associated genes, such as *Ly86* and *Aoah*, which are involved in the response to LPS, raising the possibility that maternal microbiota primes the microglia for their response to postnatal challenges. In contrast, the variation in DEGs was reversed between the sexes in adults; the number of DEGs in adult female microglia between GF and control mice was 433, but only 26 DEGs were observed in adult male microglia. Acute microbiome perturbation with antibiotic administration produced a reversed effect between the sexes in the microglia of adult mice; this effect was distinct from that in the control and GF mice (12). More DEGs were observed between the microglia of SPF and antibiotic-treated mice in males than in females. In addition, there was no significant difference in the density and morphology of microglia from antibiotic-treated and control adult mice (P60, where P0 is the day of birth). Confronted with the different effects of treatment with antibiotics and GF conditions on the microglia, Thion et al. thought that the primary effect of gut flora on microglia may have been caused by the long-term absence of the microbiota, i.e., under GF conditions. Of note, specific antibiotics utilized to clear commensal microbiota affected the microglia directly in a microbiota-independent manner; for example, minocycline is known to directly regulate microglia (75). However, the study (12) did not include detailed information regarding the antibiotics used; thus, we cannot exclude the possibility that this direct function of the antibiotics on microglia probably diminished the effect mediated by the absence of microbiota. Additionally, given that their RNA-seq data displayed several DEGs between different sexes that were dominantly present on the X and Y chromosomes, sex chromosome-associated genes may have interfered with the effects of the antibiotics on microglia, contributing to the differences between the antibiotic-treated mice and GF mice. Of note, consistent with the differences between GF and control adult mice, the DEGs in antibiotic-treated female adult mice were primarily associated with the regulation of transcription. However, DEGs in antibiotic-treated male adult mice were mainly associated with the immune response, which was distinct from GF adult male mice. Moreover, that study (12) failed to determine the specific bacterial cluster or clusters that affect microglia in the brain. It can be difficult to screen one bacterial species, given that seeding a specific species of bacteria into the host may greatly alter the composition of the host microbiota but not sole change the number of specific bacteria. However, it seems that the microbiome is unlikely to directly affect prenatal microglia, as most evidence has indicated that fetuses live in an environment devoid of any bacteria before birth, although there is a hypothesis that the first bacteria in the microbiota take root before birth (94). This indicated that indirect signaling may have occurred from intrauterine microorganisms or microbiota-generated factors from the mother via the umbilical cord during the later phases of embryonic development.

Taken together, the gut microbiota does regulate the function of microglia although the comprehensive mechanism remains uncertain. Of note, several studies revealed the potential mechanism by which the gut microbiome controls the function of microglia. Serotonin (5-hydroxytryptamine, 5-HT) is known as an important neurotransmitter in the brain and a vital factor in neurogenesis; it is primarily derived (about 90%) from the brain and the gastrointestinal system (95). Indigenous bacteria within the gut produce metabolites that signal enterochromaffin cells, which are a major producer of 5-HT in the digestive tract (16, 17). Until recently, the BBB was generally thought to be impermeable to 5-HT; however, new evidence has shown that 5-HT might cross the endothelial cells of the BBB using a serotonin transporter (15, 96). Moreover, 5-HT regulates microglial development via 5-HT2B receptors (97); therefore, it is possible that the gut microbiota affects the brain microglia via 5-HT. The gut microbiome is closely related to the maturation of microglia; several bacterial metabolites such as SCFAs may act as crucial mediators. These findings reinforce the view that the gut flora has a wide range of effects, especially on the immune response mediated by microglia in the CNS; this is supported by a study showing that differences in the microbiota composition of wild and laboratory mice modified their immune responses (98).

## The gut microbiome and microglia affect several CNS diseases

Given the importance of the microglia in the CNS, it is not surprising that microglial dysfunction results in neurodevelopmental, neurodegenerative, and neuroinflammatory diseases such as AD, PD, and MS (2–5, 88). A potential linker in this association is nitric oxide (NO) that can be generated by activated microglia (99). Indeed, during neuropathogenesis, microglia enter a hyperactive state in which they exacerbate the progression of neuropathogenesis by generating a series of factors including induced nitric oxide synthase (iNOS), chemokines, and cytokines (6). Notably, aberrant NO pathways have been implicated in several neurological disorders, including AD and PD (6). Furthermore, several microglia-associated genes are related to neurological and neuropsychiatric disorders in humans and mice, including CD33 and CXC3R1 (100–102). Commensal bacteria have emerged as a crucial factor in controlling the function and maturation of microglia; this role is mediated by the generation of SCFAs to a certain extent, as mentioned above (12, 22, 57). The host microbiota has also been found to be a critical regulator of neurogenesis, neurophysiology, myelination, and exploratory behavior (11, 81, 103, 104). The microbiome or its disruption has been shown to contribute to CNS diseases such as ASD, AD, MS, and PD, all of which are also affected by host microglia, as described below (11). Therefore, microglia that lie at the interface between environmental signals and the brain may be the critical link between the microbiome and CNS diseases.

### AD

AD is a common cognitive degenerative disease characterized by the accumulation of phosphorylated tau/tangles and amyloid plaques consisting of amyloid β (Aβ), a cleavage product of amyloid precursor protein (APP) (105, 106). Microglia are known to be related with AD and are majorly involved in the alteration of the levels of many AD-associated factors. Specifically, the phagocytic ability of microglia against Aβ aggregates can be enhanced by apolipoprotein E (ApoE), whose polymorphism has been suggested to be closely related to sporadic AD (107, 108). ApoE can be produced by microglia to some extent through regulation by peroxisome proliferator-activated receptor γ (PPARγ) and liver X receptor (LXR) (108). Both PPARγ and LXR can form heterodimers with PXR, and agonists of PPARγ and PXR can reverse some neuropathological changes and restore cognitive ability, as well as shrink the Aβ plaque burden, which depends upon ApoE and microglia (4, 108–110). Although soluble Aβ peptides and small amounts of seeded Aβ plaques are cleared by microglia under normal conditions (as mentioned above), microglia are incapable of removing excessive Aβ aggregates that chronically activate and damage both neurons and microglia during the progression of pathological Aβ accumulation (4). Triggering receptor expressed on myeloid cells 2 (TREM2), a promoter for the activation of microglia (M2-like) and phagocytosis, is also a risk factor for late-onset of AD (111). In a study identifying new susceptibility loci for AD, the microglia-associated genes *CD33* and *ABCA7* were identified as two novel susceptibility genes (100, 101).

**Figure 2:**
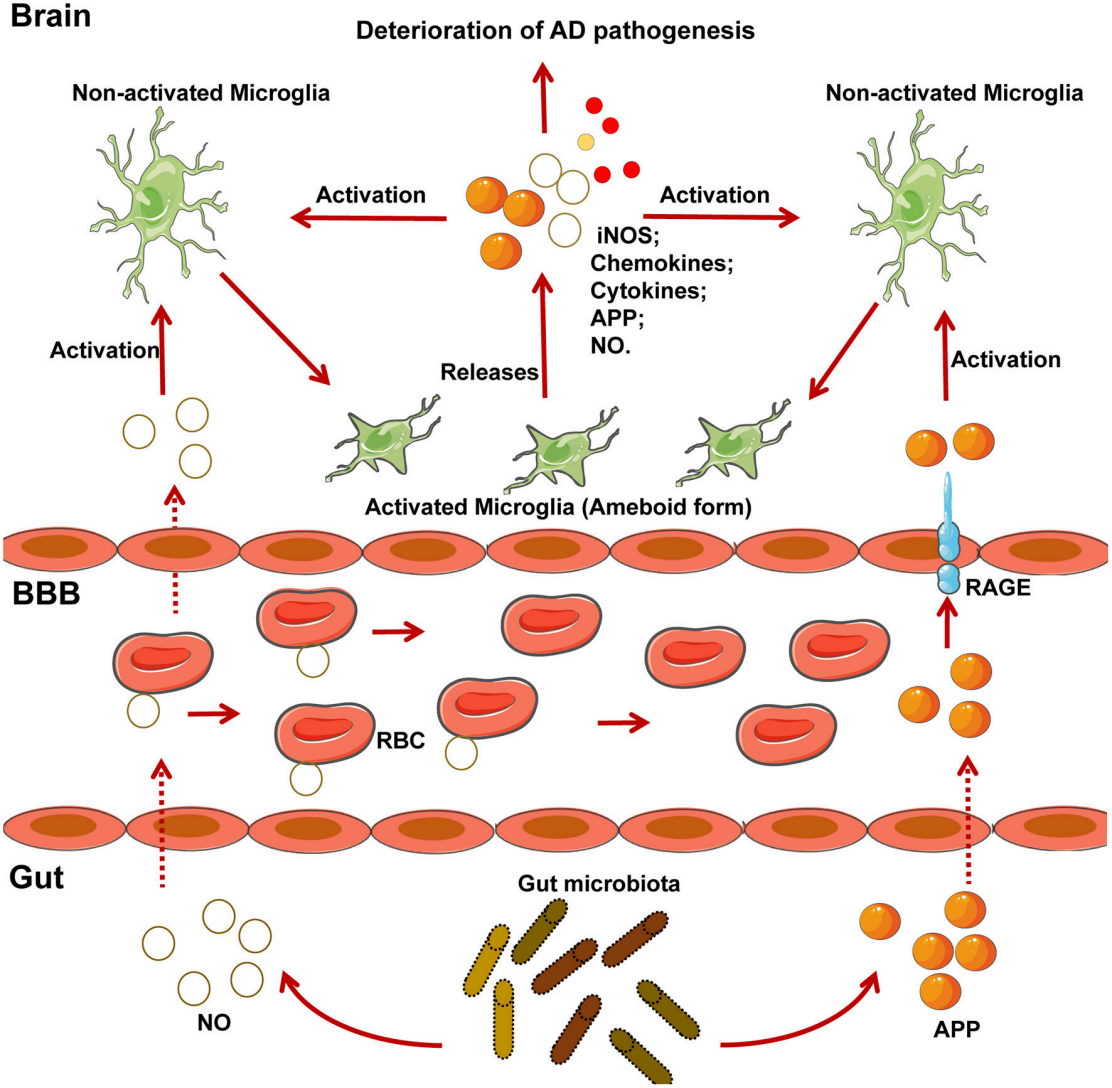
NO and APP generated by microglia and several gut-specific microbes accelerate the development of AD pathogenesis. Chronically activated microglia contribute to the progression of neurodegenerative diseases such as AD. Several gut-specific microbes are able to generate NO and APP, which activate microglia and further exacerbate the development of AD. Specifically, NO generated by gut flora, can be carried to the brain by RBC. APP secreted by gut flora can cross the BBB via the RAGE receptor. After reaching the CNS, both NO and APP activate the microglia, which exhibit an ameboid form. Activated microglia can then secrete several risk factors for AD, including iNOS, chemokines, cytokines, APP, and NO, which further accelerate the pathogenesis of AD. iNOS, inducible nitric oxide synthase; NO, nitric oxide; RBC, red blood cell; RAGE, receptor for advanced glycosylation products.

The gut microbiome is also closely related to AD in mice model and humans, although the detailed regulatory mechanisms surrounding this association require further research (6, 112–115). Different gut bacteria, such as *Escherichia coli, Salmonella enterica, S. typhimurium, Bacillus subtilis, Mycobacterium tuberculosis*, and *Staphylococcus aureus* (6), generate a significant concentration of amyloids and LPS (116). *E. coli* endotoxin potentiates the formation of Aβ *in vitro*, and as Aβ exceeds a certain threshold, it self-propagates, both of which may further compromise CNS function (6). As a result of aging, the gastrointestinal epithelium and BBB are more permeable to small molecules, which is exacerbated by alterations in tight junctions that result from changes in the gut microbiota when *Bacteroidetes* outnumbers *Firmicutes* and *Bifidobacterium* (6). From such perspective, it is reasonable that the migration of amyloids and leukocytes from the gut to the brain is more prominent in aging individuals. Notably, amyloids can activate the microglia to cause prolonged inflammation (116, 117). Of note, activated microglia can produce APP in response to exhausted neuronal excitation by NO and APP from the gut, which further contributes to AD pathogenesis (116) (Figure 2). APP overexpression is associated with the upregulation of peroxynitrite, which can transform into NO, and activated microglia also produce high concentrations of inducible NO synthase (Figure 2); this supports the hypothesis that NO generated by activated microglia is closely related to Aβ deposition (99). Importantly, the gut is the main site of NO generation, and the generation of high concentrations of NO is mediated by several bacterial species (Figure 2). For example, gut *lactobacteria, bifidobacteria*, and *E.coli Nissle 1917* can generate NO through the conversion of nitrite and nitrate (6). Moreover, NO produced in the gastrointestinal system can be scavenged by hemoglobin in red blood cells, thereby entering the peripheral vasculature and crossing the BBB into the cerebral vasculature (118), initiating a vicious cycle, as demonstrated in Figure 2.

Given that the gut flora controls the maturation and function of microglia (12, 22), it is likely that microglial activation controlled by gut microbiota could be strongly associated with AD. Indeed, the proportion of *Allobaculum* and *Akkermansia* in the gut was found to be reduced in an APP/PS1 mouse model of AD, along with an increase in the proportion of *Rikenellaceae* (112). Of note, colonization of GF APP transgenic mice by the microbiota from conventionally-raised APP transgenic mice through the fecal route increased cerebral Aβ pathology (113). AD occurs in the elderly and aged individuals exhibit a different microbiome, which supports the possibility that altered microbiome mediated by aging is associated with the initiation and development of AD. Additionally, another study confirmed the modulation of microglia through external vagus nerve stimulation in a murine model of AD (88) (Figure 2). However, whether the microbiota affects the progression of AD through several bacterial species or their associated factors remains unresolved.

### MS

**Figure 3:**
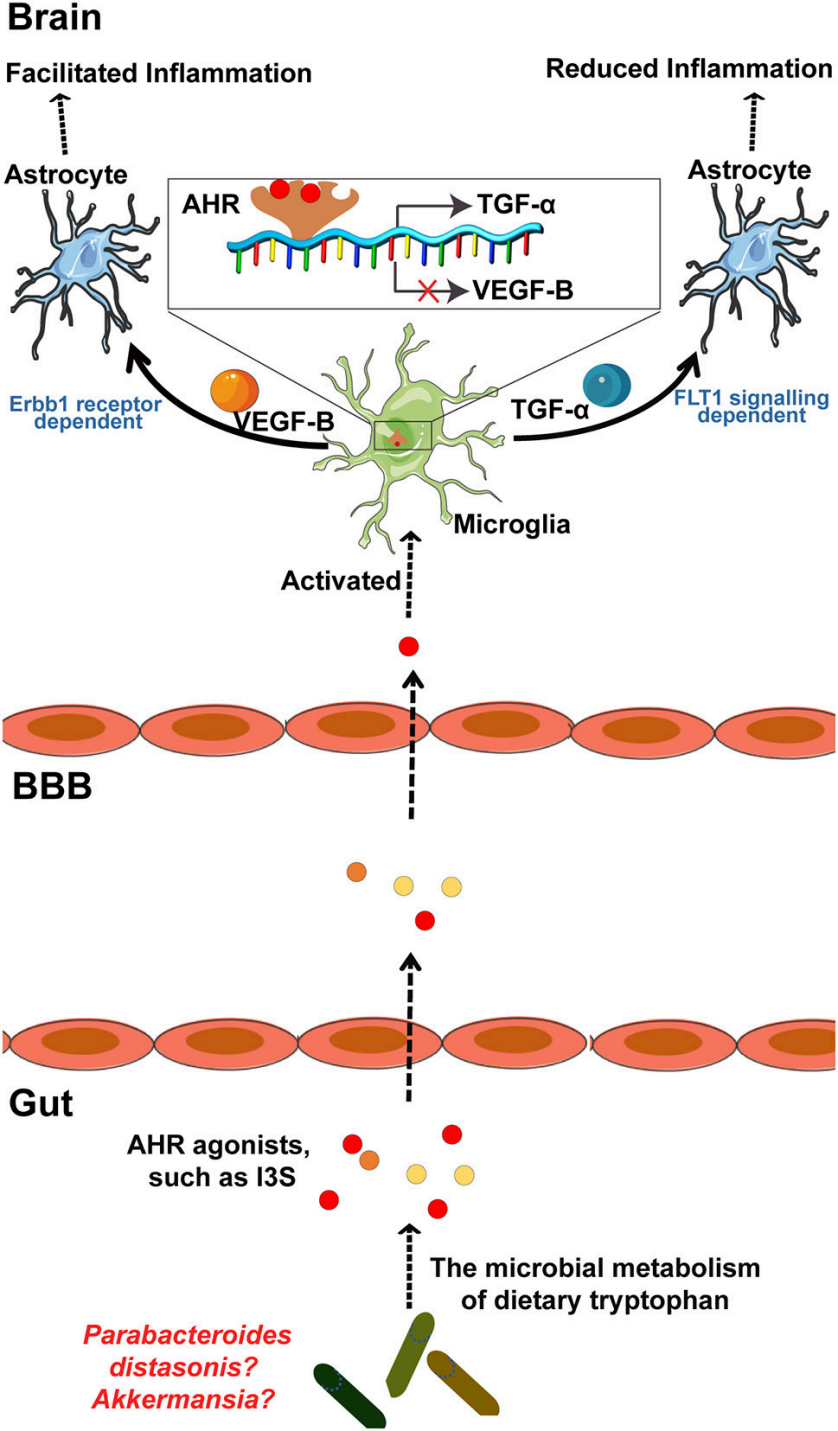
The microbial metabolism of dietary Trp regulates the inflammatory response of astrocytes by microglia in EAE mouse model of MS through the AHR generated within microglia. Metabolism of dietary Trp by microbiota generates AHR agonists, which cross the BBB into the brain to activate microglia through an AHR-mediated mechanism within the microglia. In detail, these AHR agonists function as ligands for the AHR expressed in the microglia to bind the genes encoding VEGF-B and TGF-α, as indicated, to facilitate TGF-α transcription and to inhibit VEGF-B expression. Notably, VEGF-B increases the inflammatory activation of astrocytes to control the development of EAE. In contrast, TGF-α weakens the inflammatory activation of astrocytes to worsen EAE. Of note, both of *Akkermansia* and *Parabacteroides* distasonis, MS associated microbiota, may be the producer of AHR agonist, which required to be further researched. AHR, aryl hydrocarbon receptor; Trp, tryptophan; MS, multiple sclerosis; EAE, experimental autoimmune encephalomyelitis.

MS is a chronic inflammatory demyelinating disease of the CNS with heterogeneous histopathological features (119–121). In an EAE rat model of MS, relapse was characterized by an imbalance of monocyte activation profiles toward the M1 phenotype and the suppression of immunomodulatory M2 macrophages and/or microglia at lesion sites (122, 123). In addition, several microglia-associated genes, such as *TNFRSF1A* and *IRF8*, were also found to be associated with MS (122, 124). Moreover, TGFα and VEGF-B produced by microglia modulate the pathogenic activities of astrocytes in the EAE mouse model of MS and humans (23). In detail, TGFα acts via the Erbb1 receptor and suppresses the pathogenic activities of astrocytes to limit EAE development, whereas VEGF-B activates FLT-1 signaling in astrocytes and worsens EAE (Figure 3). More specifically, metabolites of dietary tryptophan, generated by gut microbiota, modulate the production of TGFα and VEGF-B by microglia through an aryl hydrocarbon(AhR) receptor-mediated mechanism to further regulate CNS inflammation (23).

Commensal bacteria can also affect the autoreactivity of peripheral immune cells to CNS self-antigens, and thus, great attention has been focused on the contribution of the microbiota to MS pathogenesis (69, 120, 121, 125). Moreover, there is a significant increase in some taxa such as *Akkermansia* and reduction in other taxa such as *Parabacteroides* distasonis in untreated MS patients, while whether these bacteria regulate the microglia mediated inflammatory response through a mechanism by which the metabolites of tryptophan AhR ligands remains uncertain (Figure 3). However, AhR ligands are solely produced by a few bacteria, such as *Peptostreptococcus* russellii and *Lactobacillus* spp (126). Moreover, microbiota transplants from MS patients into GF mice resulted in worsening the symptoms of EAE compared with those in mice transplanted with microbiota from healthy controls (121). Additionally, on transplanting the microbiota to a transgenic mouse model of spontaneous brain autoimmunity, MS twin-derived microbiota induced a significantly higher incidence of autoimmunity than the healthy twin-derived microbiota (120). A majority of MS patients harbor antibodies against certain gastrointestinal antigens that are not found in healthy individuals, which further supports the possibility that alterations in the immune status of the brain against gut microbiota may be associated with MS (127). In an experimental model of MS, GF mice exhibited complete protection, which may have resulted from attenuated Th17 and B cell responses (128). The short-term effect of microbiota removal by treatment with antibiotics also attenuated EAE progression by inducing T cell, B cell, and immature natural killer (iNK), and regulatory T cell (Treg) responses (125, 129).

The close relationship between the gut microbiota and MS was also found in Theiler's murine encephalomyelitis virus (TMEV)-induced demyelinating disease (IDD), another relevant MS model (57). Unlike the effect showed by the gut microbiome exhibited in the previously mentioned MS model, antibiotic administration exaggerated the progression and severity of TMEV-IDD. A study into the mechanisms of this effect revealed that antibiotic-treated mice displayed lower levels of CD4^+^ and CD8^+^ T cells in their cervical and mesenteric lymph nodes. Furthermore, the activation of microglia was enhanced in TMEV-infected mice following treatment with antibiotics. Collectively, microglia act as crucial mediators in the connection between the microbiome and MS. However, the detailed mechanism underlying gut microbiota regulation of MS remains uncertain.

### Depression

Despite the abundance of research on the mechanisms of depression as a psychiatric disease, our current understanding of depression is limited. One hypothesis claims that depression is a microglial disorder because microglial dysfunction has been commonly observed in the brain at the onset of depression (25). Minocycline, an antibacterial agent known to inhibit the activation of microglia after crossing the BBB, has been shown to significantly diminish depressive behaviors in rodents and humans (130, 131). Given that the gut microbiome affects the maturation of microglia, it is not clear whether the effect of minocycline is due to its antimicrobial properties by destroying gut microbiota or due to the direct inhibition of microglia. Microglia exhibit varying degrees of activation in patients with schizophrenia and depression, especially those who are suicidal (132). This was also confirmed in animal models of depression, although the pathophysiological role of microglial activation has not been clearly confirmed (25, 132–134). Studies of rodent models showed that the gut microbiome is also involved in the development of depression (26–29), and beta diversity of the gut microbiome was significantly different between depressed and control patients. Of note, administration of *Bifidobacterium infantis* reversed experimentally created anxiety and depression in GF mice (26), and *Bifidobacterium infantis* is defined as a “psychobiotic” because of its antidepressant effect (135). GF mice transplanted with fecal microbiota from depressed mice also exhibited depressive-like phenotypes, which was confirmed in a rat depression model (29, 136). Treatment with antibiotics during the first year of life is also closely correlated to depression later in the life of humans (137). Probiotic supplementation was shown to improve the symptoms of anxiety and depression, especially *Lactobacillus helveticus Ns8*, which improved the behavioral, cognitive, and biochemical aberrations caused by chronic restraint stress (138, 139). However, the detailed mechanisms behind this association remain unclear, especially whether these results were mediated by the known interaction between the gut microbiome and microglia, direct effect of other unknown factors generated by gut bacteria, or a combination of the two.

### PD

PD, a progressive neurodegenerative disease with motor and non-motor symptoms, is associated with misfolded α-synuclein seeds that assemble into fibrillary inclusions and with the loss of dopaminergic neurons in the substantia nigra (4, 140). α-synuclein, generated from neuron-derived exosomes, triggers the activation of microglia, which may accelerate lethality by the TAM-dependent phagocytosis of distressed spinal motor neurons (9). The mice lacking CX3CR1, a microglial-associated inflammation factor, exhibited a higher loss of neuron cells than control mice in a PD model established by 1-methyl-4-phenyl-1,2,3,6-tetrahydropyridine (141).

From the perspective of gut microbiology, PD patients exhibited significantly different microbial populations compared with healthy controls (142–144). PD patients displaying the tremor-dominant phenotype had significantly lower *Enterobacteriaceae* than those with more severe postural and gait instability, suggesting that the relative abundance of specific bacteria is sufficient to distinguish between the different forms of PD (143). Several microbial molecules that mimic host structures promote an immune response during the development of AD and PD (145–147). Of note, gut microbiome regulates movement disorders in mice and alterations in the human microbiome represent a risk factor for PD (140). In detail, antibiotic treatment ameliorates while microbial re-colonization promotes the pathophysiology of PD in adult animals. Administration of specific microbial metabolites to GF mice promotes neuroinflammation, and colonization of α-Syn-overexpressing mice with microbiota from PD-affected patients enhances physical impairments compared to microbiota transplants from healthy humans (140). However, given that there is no definite association among microglia, microbiota, and PD, the detailed underlying mechanisms remain uncertain.

### ASD

ASD, a disorder associated with neurodevelopmental difficulties and mood disorders, is also associated with microglial dysfunction. Current understanding of the role of microglia in ASD is focused on the excessive microglia-mediated loss of synaptic tissue, which has been shown to contribute to patients suffering from Rett syndrome, an X-linked form of ASD (148). The gut microbiome in ASD patients exhibited differences in species variety and complexity compared with neurotypical controls (149–156). Generally, the gut microbiome of ASD patients exhibited more diversity and an abundance of *Clostridia* species (149–151, 155), but lacked several beneficial bacterial populations, such as *Prevotella* (154). In addition, *Sutterella* was found in intestinal biopsies of children with ASD presenting with gastrointestinal symptoms, while it was absent in control samples (152). Moreover, microbiota transfer therapy (MTT) alters the gut ecosystem and significantly improves GI and behavioral symptoms of ASD (157). Overall bacterial diversity and the abundance of *Bifidobacterium, Prevotella*, and *Desulfovibrio* among other taxa increased following MTT. Collectively, these studies indicate a potential relationship between the microbiome (or specific microbes) and the brain in ASD patients; as to whether microglia function as a mediator in this relationship remains uncertain. However, augmenting the microbiome with a specific microbe may be beneficial for ASD patients.

### Abnormal behavior

Microglial dysfunction is also closely associated with host abnormal behavior(13, 102, 158). Indeed, microglia interact with the nervous and endocrine systems during development, in which prostaglandin E2 (PGE2), generated through the aromatization of estradiol, plays a role (158). Additionally, mice lacking microglia-specific genes, such as CX3CR1, a chemokine receptor, exhibited impaired brain connectivity and abnormal social behavior because of defective neuron-microglia signaling (102). Of note, modifying the host microbiome with antibiotic or probiotic treatment also has a profound effect on the development of individuals, with long-lasting effects on host behavior in humans and animals (137, 159–161). Antibiotic administration during the first year of life may result in negative neurocognitive outcomes in later phases of life, including behavioral difficulties (137). Additionally, another study indicated that 7-day administration of nonabsorbable antibiotics was adequate to decrease anxiety-like behavior in mice (21). In contrast, short-term treatment with vancomycin failed to lead to anxiety- and depressive-like behaviors in neonate rats (16, 162), suggesting that the effect of gut dysbiosis on host behavior varies in different animals and depleting the microbiome in the short-term may not always impact behavior (162–164). In addition, compared to SPF mice, GF mice displayed several phenotypes associated with behavior (10, 21, 165–169). Moreover, several bacterial species improved the symptoms of stress, anxiety, and depressive-like behavior, as well as facilitating improvements in social behavior, communication, and cognitive function in animal models (13, 27, 163, 165, 170–174). Notably, several probiotic strains in rodents produced behavioral effects in a vagus nerve activation-dependent manner (175). Given that the vagus nerve also regulates the function of microglia at specific disease states, it may play a crucial role in the gut-microglia connection in abnormal behavior (Figure 2).

Collectively, the gut-microglia connection is clearly implicated in many CNS diseases, although the detailed mechanisms controlling many of these associations remain to be determined. Of note, CNS disease-associated microRNAs have been shown to be closely related to the gut microbiome (176). MicroRNAs act as translational repressors to regulate gene translation and have also been implicated in anxiety-like behaviors (176). A previous study revealed that the gut microbiome regulates microRNA expression in the amygdala, affecting factors such as miR-183-5p and miR-206-3p, both of which are implicated in influencing anxiety levels and the expression of neurotrophins such as brain-derived neurotrophic factor (176). Moreover, in a study identifying region-specific miRNA-mRNA networks in the dorsal raphe nucleus and amygdala of high-responder/low-responder rats, miR-206-3p was shown to be associated with microglia and the immune response (177). Collectively, microRNAs may be potential mediators in the function of the gut-microglia connection in CNS diseases. In addition, in relation to the gut-microglia connection, HSV has clinically been associated with several neurodegenerative diseases, such as AD and MS, with uncertain mechanisms (106, 178). Of note, as a neurotropic virus, HSV-1 was also found to infect enteric neurons in addition to affecting the CNS, leading to gut dysbiosis (179). This may increase the abundance of several NO- and APP-generating bacteria, which remains uncertain. Consequently, NO and APP cross the BBB to activate microglia, thereby accelerating the progression of CNS diseases (Figure 2); HSV-1 may, therefore, be considered as a chronic risk factor for the development of several neurodegenerative diseases, especially AD (178).

## Conclusions and future directions

Research over the past few years has revealed that the development of a healthy brain requires crucial pre- and post-natal events that integrate environmental cues, especially the gut microbiome (11). The gut microbiome has been shown to influence various aspects of CNS biology through multiple mechanisms, including the alteration of both neurotransmitter levels (17) and BBB permeability (18). Furthermore, gut microbiome is closely associated with CNS diseases such as AD, depression, PD, and even ASD (11). However, most research to date has only demonstrated that different proportions of bacteria genera are associated with several CNS diseases; our understanding of the detailed signaling pathways through which the microbiome modulates CNS diseases remains poor. Importantly, microglia may be the crucial mediators linking gut microbiome and CNS diseases, given that microglia are crucial immune cells in the CNS, and their dysfunction has been shown to be related to most CNS-associated diseases. Moreover, emerging studies have revealed that the gut microbiome controls the maturation and function of microglia (12, 22) although the detailed mechanisms are yet to be elucidated. Indeed, as summarized by this study, multiple CNS diseases, such as AD, PD, and ASD, that are closely associated with microglia are also related to the gut flora. Additionally, indirect evidence supports the notion that the microbiota may affect the brain by modulating the microglia. For example, prostaglandin D2, which is produced by microglia, acts on the DP1 receptor in astrocytes to induce astrogliosis and demyelination (180). Microglia are known to participate in the demyelination process and the gut microbiome is also involved in myelination because myelin-related transcripts were found to be increased in the prefrontal cortex of the brains of GF mice and antibiotic-treated mice as compared to control mice (181, 182). However, the possibility that the gut microbiome-mediated effect on myelination is associated with microglia cannot be excluded and requires further research. Furthermore, just as several CNS diseases have shown significant sexual bias—such as ASD, which affects males at a higher rate (183, 184)—the composition of the gut microbiome and microglia also exhibits sexually dimorphic properties. Thus, it is essential to determine whether the gut-microglia connection contributes to the progression of CNS diseases with a sex bias. It is possible that the microbiome affects CNS diseases by modulating microglia; however, extensive research is required to confirm this. Therefore, it is critical to determine the importance of the gut-microglia connection in CNS diseases and to clearly define the specific mechanisms involved. Furthermore, understanding the interactions between specific microbial species and microglia would be significant for developing a novel therapeutic strategy for the treatment of neurological disorders in humans. Recently, the Kallyope company was allowed to continue its work on harnessing the communication pathways between the gut and the brain to develop novel therapies for various illnesses of the CNS. However, further studies are required to determine whether probiotics can be exploited to improve microglial function and, ultimately, alleviate CNS diseases.

## Author contributions

YilW conception, design, collection and/or assembly of references, discussion, interpretation, and manuscript writing. ZW and YuW collection and/or assembly of references, interpretation, and manuscript writing. FL, XS, JJ, SQ, and RW collection and/or assembly of references. FJ, KK, and YifW conception, design, interpretation, and final approval of manuscript. YilW, ZW, and YuW contributed equally to this article.

### Conflict of interest statement

The authors declare that the research was conducted in the absence of any commercial or financial relationships that could be construed as a potential conflict of interest.
